# Chronic ethanol exposure of human pancreatic normal ductal epithelial cells induces cancer stem cell phenotype through SATB2

**DOI:** 10.1111/jcmm.13666

**Published:** 2018-05-15

**Authors:** Wei Yu, Yuming Ma, Sharmila Shankar, Rakesh K. Srivastava

**Affiliations:** ^1^ Kansas City VA Medical Center Kansas City MO USA; ^2^ Department of Pathology School of Medicine University of Missouri‐Kansas City Kansas City MO USA; ^3^ Department of Pharmaceutical Sciences University of Missouri‐Kansas City Kansas City MO USA; ^4^ Present address: Department of Genetics Stanley S. Scott Cancer Center Louisiana State University Health Sciences Center New Orleans LA USA

**Keywords:** alcohol, cancer stem cell, pancreatic cancer, pluripotency, self‐renewal, transformation

## Abstract

The incidence of pancreatic cancer is on the rise. Risk factors for pancreatic cancer include alcohol toxicity and metabolic conditions such as obesity, hypertension, dyslipidaemia, insulin resistance and type 2 diabetes. However, the molecular mechanism by which chronic alcohol consumption contributes to pancreatic cancer is not well understood. The purpose of the study was to demonstrate the effects of long‐term chronic ethanol exposure on the transformation of human pancreatic normal ductal epithelial (HPNE) cells. Our data showed that ethanol‐transformed HPNE cells were more progressively transformed exhibiting spheroids and colonies, and anchorage‐independent growth. These transformed cells contained high levels of reactive oxygen species and induced SATB2 expression. Furthermore, during ethanol‐induced cellular transformation, cells gained the phenotypes of cancer stem cells (CSCs) by expressing pluripotency maintaining factors (Oct4, Sox2, cMyc and KLF4) and stem cell markers (CD24, CD44 and CD133). Ethanol‐induced SATB2 can bind to the promoters of KLF4, Oct4, cMyc, Sox2, Bcl‐2 and XIAP genes. Suppression of SATB2 expression in ethanol‐transformed HPNE cells inhibited cell proliferation, colony formation and markers of CSCs and pluripotency. These data suggest that chronic alcohol consumption may contribute toward the development of pancreatic cancer by converting HPNE cells to cancer stem‐like cells.

## 1 INTRODUCTION

Pancreatic cancer has an exceptionally high mortality rate and is the fourth leading cause of cancer‐related death in the United States.^1^ With an overall 5‐year survival rate of 6%,^2^ pancreatic cancer has one of the poorest prognoses among all cancers.^3^ The incidence of pancreatic cancer varies significantly across regions, which suggests that several factors may be responsible for this deadly disease.^4^ The genetic, race, gender, environmental carcinogen, diet and lifestyle appears to be the primary factors for pancreatic cancer.^5^ Other factors such as smoking, alcohol, coffee consumption and exposure to organochlorine or hydrocarbon solvent have been associated with the frequency and spectrum of K‐ras mutation in pancreatic tumours.^6^, ^7^, ^8^, ^9^ Metabolic conditions such as obesity, hypertension, dyslipidaemia, insulin resistance, and type 2 diabetes are also the risk factors for pancreatic cancer.^8^, ^10^ About 5%‐10% of patients with pancreatic cancer have underlying germline mutations or disorders, whereas the remaining percentage of cancer cases may be because of somatic mutations.^4^

Epidemiological data strongly suggest that the heavy alcohol drinking increases the risk for pancreatic cancer.^11^, ^12^, ^13^, ^14^ A recent study has demonstrated that chronic alcohol intake promotes intestinal tumorigenesis and tumour invasion in genetically susceptible mice, increases in polyp‐associated mast cells, and mast‐cell‐mediated tumour migration in vitro,^15^ suggesting mast‐cell‐mediated inflammation could promote carcinogenesis.^15^ These data confirm that ethanol and its metabolites are the potential human carcinogen. However, the molecular mechanism by which ethanol toxicity induces malignant transformation of human pancreatic normal ductal epithelial (HPNE) cells is not known.

SATB2 (special AT‐rich binding protein‐2), a transcription factor and epigenetic regulator that binds DNA 16 to regulate gene expression.^17^, ^18^, ^19^ SATB2 gene is required for normal mammalian development; however, it is not expressed in healthy adult cells. SATB2 is essential for proper facial patterning of the embryo and healthy bone development.^19^ Inappropriate activation of this gene may be the cause of malignant cellular transformation. SATB2 regulates transcription of pluripotency maintaining factors (Sox2, cMyc, KLF4 and Oct4) which form the core regulatory positive feedback‐loop to sustain self‐renewal capacity of stem cells. Using chromatin immunoprecipitation assay, we have shown that SATB2 can directly bind to the promoters of Bcl‐2, Bsp, Nanog, cMyc, XIAP, KLF4 and Hoxa2, suggesting a role of SATB2 in the regulation of cell survival, pluripotency and proliferation.^20^ Therefore, we reasoned to believe that SATB2 proteins may play a critical role during chronic ethanol exposure of human pancreatic ductal epithelial cells.

The primary goal of this paper was to examine the molecular mechanisms by which chronic ethanol exposure induces cellular transformation of HPNE cells which may lead to pancreatic carcinogenesis. To investigate the role of SATB2 at an early step of cell transformation, we utilized HPNE cells as a model to generate progenitor cells by chronic ethanol exposure. Our studies have established a novel link between exposure to ethanol and SATB2‐regulated transformation of HPNE cells.

## 2 MATERIALS AND METHODS

### 2.1 Cell culture conditions and reagents

Human pancreatic normal ductal epithelial cells were purchased from American Type Culture Collection, Manassas, VA. HPNE cells were grown in well‐defined cell medium as described.^21^ Antibodies against SATB2 and β‐actin were purchased from Abcam (Cambridge, MA). Enhanced chemiluminescence (ECL) Western blot detection reagents were purchased from Amersham Life Sciences Inc. (Arlington Heights, IL).

### 2.2 Cell proliferation assay

Cells (1.5 × 10^4^) were incubated for various time points in 1 mL of culture medium. Cell viability was determined by trypan blue assay using Countess™ Automated Cell Counter (Invitrogen).

### 2.3 Colony formation assay

Colony formation assays were performed as described elsewhere.^22^ In brief, cells were seeded into 6‐well plates at a low density (200 cells per well). Cell culture medium was renewed every 3 days. After 21 days, colonies were fixed with cold methanol and then stained with 0.5% crystal violet. The colonies were imaged with a microscope.

### 2.4 Spheroid formation

Spheroids formation assays were performed as described elsewhere.^22^ In brief, cells were plated in ultra‐low attachment plates at a density of 100‐500 cells/mL. The spheroid formation in suspension was measured after 10 days of culture using a Nikon Eclipse microscope (Nikon).

### 2.5 Lentiviral particle production and transduction

The lentivirus production and transduction were performed as described elsewhere.^22^ In brief, lentivirus was produced by triple transfection of HEK 293T cells. Packaging 293T cells were plated in 10‐cm plates at a cell density of 5 × 10^6^ 1 day before transfection in DMEM containing 10% heat‐inactivated foetal bovine serum. 293T cells were transfected with 4 μg of plasmid and 4 μg of the lentiviral vector using lipid transfection (Lipofectamine‐2000/Plus reagent; Invitrogen) according to the manufacturer's protocol. Viral supernatants were collected and concentrated by adding PEG‐it virus precipitation solution (SBI System Biosciences) to produce virus stocks with titres of 1 × 10^8^‐1 × 10^9^ infectious units per mL. Viral supernatant was collected for 3 days by ultracentrifugation and concentrated 100‐fold. Titres were determined on 293T cells. Cells were transduced with lentiviral particles expressing the gene of interest.

### 2.6 Western blot analysis

The Western blot analysis was performed as we described earlier.^23^ In brief, cell lysates were subjected to SDS‐PAGE, and gels were blotted onto nitrocellulose membrane (Amersham Biosciences, Piscataway, NJ, USA). The membranes were blocked with 5% BSA in Tris‐Tween buffered saline at 37°C for 2 hours and then incubated with primary antibody diluted in Tris‐buffered saline (1:1000 dilutions) overnight at 4°C, with gentle shaking. The membranes were then washed 3 times with Tris‐buffered saline‐T (TBS‐T) and incubated with the secondary antibody linked to horseradish peroxidase (1:5000) for 1 hour. After incubation with secondary antibody, the membranes were rewashed 3 times with TBS‐T. Finally, protein‐antibody complexes were detected by the addition of ECL substrate (Thermo Fisher Scientific, Rockford, IL).

### 2.7 Chromatin immunoprecipitation assay

Chromatin immunoprecipitation (ChIP) assays were performed as we described elsewhere.^24^, ^25^ In brief, chromatin was immunoprecipitated using anti‐SATB2 antibody. Normal rabbit IgG (Abcam) was used as a negative control. ChIP‐derived DNA was measured by 2% agarose gel electrophoresis.

Bcl‐2 promoterChIP‐F, TTTCAGCATCACAGAGGAAG
Bcl‐2 promoterChIP‐R, CAATCACGCGGAACACTTGATT
Oct4‐ChIP‐F, ATGACCACTGCGCCCGGACTGC
Oct4‐ChIP‐R, ACTTGGATCTCTTCCAAGTGC
KLF4‐Chip‐F, ACCGGACCTACTTACTCGCC
KLF4‐Chip‐R, TCGGCAGCCCGAAGCAGCTGG
cMyc‐Chip‐F, AATTAATGCCTGGAAGGCAGCC
cMyc‐ChIP‐R, AGTCAGCAGAGACCCTTGTG
XIAP‐Chip‐F,TCCAAGAGAGATGCACTAGGGTC
XIAP‐Chip‐R, TTATGGCAAGATCTATGTGGAACTC

### 2.8 Quantitative real‐time PCR

Total RNA in cells was extracted by the TRIzol reagent (Invitrogen) and reverse transcribed into cDNA using High‐Capacity cDNA Reverse Transcription Kit (Thermo Fisher Scientific). qRT‐PCR was conducted using fast SYBR Green Master Mix (Thermo Fisher Scientific). The 2^−ΔΔ*Ct*^ method was used to evaluate relative mRNA expressions compared with controls. The following gene‐specific primers were used:

Sox2 (5′‐AAC CCC AAG ATG CAC AAC TC‐3′, 5′‐GCT TAG CCT CGT CGA TGA AC‐3′)
cMyc (5′‐CGA CGA GAC CTT CAT CAA AA‐3′, 5′‐TGC TGT CGT TGA GAG GGT AG‐3′)
Oct4 (5′‐GGA CCA GTG TCC TTT CCT CT‐3′, 5′‐CCA GGT TTT CTT TCC CTA GC‐3′)
CD24 (5′‐ATG GGA ACA AAC AGA TCG AA‐3′, 5′‐TTT GCT CTT TCA GCC ATT TC‐3′)
CD44 (5′‐ACT TCA CCC CAC AAT CTT GA‐3′, 5′‐GTG GCT TGT TGC TTT TCA GT‐3′)
CD133 (5′‐CCT CTG GTG GGG TAT TTC TT‐3′, 5′‐CCT CTG GTG GGG TAT TTC TT‐3′)
HK‐GAPD (5′‐GAG TCA ACG GAT TTG GTC GT‐3′, 5′‐TTG ATT TTG GAG GGA TCT CG‐3′)

### 2.9 Statistical analysis

The mean and SD were calculated for each experimental group with replicates. Differences between groups were analysed by ANOVA, followed by Bonferroni's multiple comparison tests using PRISM statistical analysis software (GrafPad Software, Inc., San Diego, CA). Significant differences among groups were calculated at *P* < .05.

## 3 RESULTS

### 3.1 Ethanol induces transformation of HPNE cells by up‐regulating SATB2 expression

**Figure 1:**
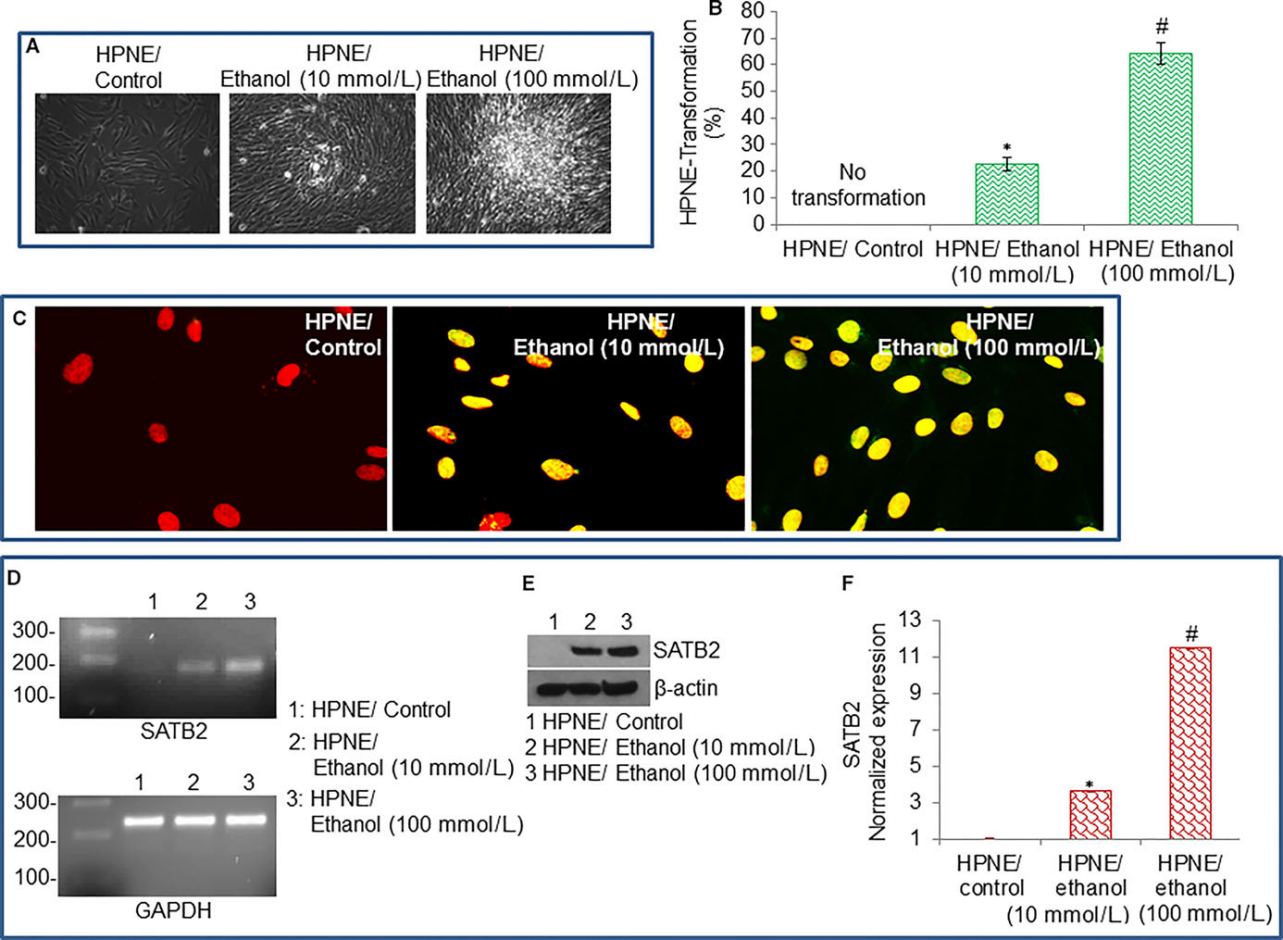
Chronic ethanol exposure induces human pancreatic normal ductal epithelial (HPNE) cell transformation by inducing SATB2 expression. A, Transformation of HPNE cells. Phase contrast imaging of HPNE/Control, and ethanol‐transformed HPNE (HPNE/Ethanol) cells. HPNE cells were grown in the well‐defined culture medium as per American Type Culture Collection recommendations. HPNE cells were cultured for 6 mo with 2 different concentrations of ethanol (10 and 100 mmol/L). Photographs were taken under phase contrast microscope. B, HPNE cell transformation efficiency. Data represent mean ± SD. *, #Significantly different from control, *P* < .05. C, Expression of SATB2 by immunohistochemistry (IHC). IHC was performed to examine the nuclear expression of SATB2 in HPNE/Control and HPNE/Ethanol cells as we described elsewhere.^22^ Red colour = nucleus. Yellow colour = red (nucleus) + green (SATB2) = merged picture (expression of SATB2 in nucleus). D–F, SATB2 expression in HPNE/Control and HPNE/Ethanol transformed cells was measured by PCR, Western blot analysis, and qRT‐PCR, respectively. qRT‐PCR data represent mean ± SD. *, #Significantly different from HPNE/Control cells, *P* < .05

We have used HPNE cells as a model to assess whether chronic ethanol exposure induces malignant transformation. HPNE cells were grown in culture medium in the presence or absence of ethanol (10 and 100 mmol/L) for 6 months. Long‐term chronic exposure of HPNE cells to ethanol‐induced cellular transformation as evident by the formation of clumps, loss of contact inhibition, and disoriented growth (Figure 1A). HPNE cell transformation efficiency was significantly higher with the higher dose of ethanol (100 mmol/L) compared to 10 mmol/L ethanol exposure (Figure 1B).

SATB2 plays a vital role in the chromatin remodelling and regulation of genes which participates in cell growth, survival, differentiation, self‐renewal and pluripotency. We, therefore, examined the mechanism of ethanol‐induced transformation of HPNE cells by comparing the expression of SATB2 in HPNE control cells and ethanol‐transformed HPNE cells (HPNE/Ethanol). As shown in Figure 1C‐E, 6‐month exposure of HPNE cells to ethanol‐induced the expression of SATB2 gene as measured by immunohistochemistry, polymerase chain reaction, Western blotting and quantitative real‐time polymerase chain reaction. SATB2 was not expressed in normal HPNE cells. By comparison, SATB2 was expressed in the nuclei of HPNE/Ethanol cells (appearance of yellow colour), but not in HPNE/Control cells. Short‐term exposure (up to 1 month) of HPNE cells to ethanol did not induce SATB2 (data not shown). Our data suggest that ethanol can induce HPNE cell transformation which is associated with the induction of SATB2.

### 3.2 Ethanol‐transformed HPNE cells form spheroids in suspension and colonies in soft agar, express stem cell markers and pluripotency maintaining factors, and generate reactive oxygen species

**Figure 2:**
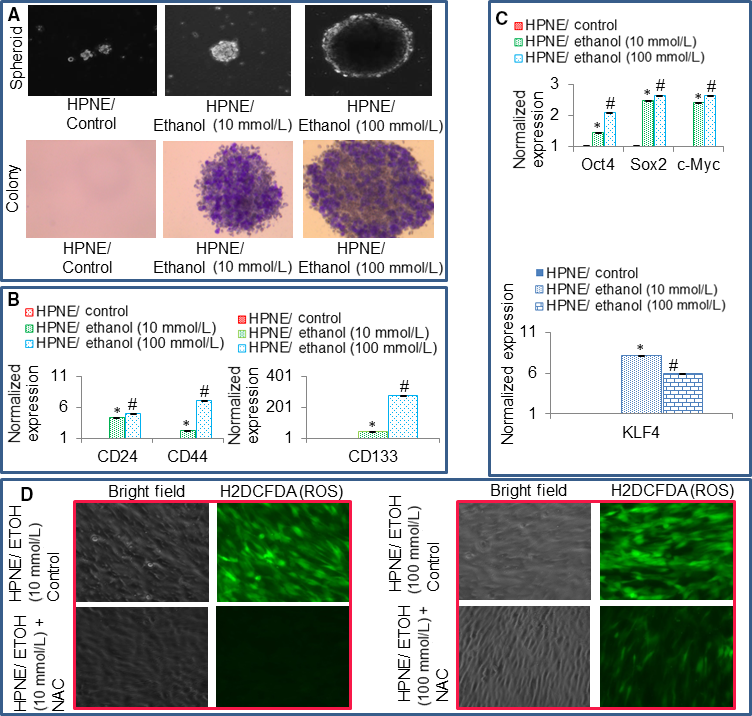
Formation of spheroids and colonies, and induction of stem cell markers and pluripotency maintaining factors by ethanol‐transformed human pancreatic normal ductal epithelial (HPNE) cells. A, Spheroid and colony formation. Normal HPNE (HPNE/Control) and ethanol‐transformed HPNE (HPNE/Ethanol) cells after 6‐mo of exposure with ethanol were grown in either suspension for 10 d for spheroid formation or in soft agar for 21 d for colony formation. Photographs were taken. B, Expression of stem cell markers. RNA was extracted from cells, and qRT‐PCR analysis was performed to measure the expression of CD24, CD44 and CD133. GAPDH was used as an internal control. C, Expression of pluripotency maintaining factors. RNA was extracted, and qRT‐PCR analysis was performed to measure the expression of Oct4, Sox2, cMyc and KLF4 as we described elsewhere.^28^, ^49^ GAPDH was used as an internal control. Data represent mean ± SD. *, #Significantly different than HPNE/Control cells, *P* < .05. D, Generation of reactive oxygen species (ROS) by ethanol‐transformed‐HPNE cells. Ethanol‐transformed HPNE cells were treated with or without *N*‐Acetyl cysteine (NAC) for 8 h and stained with H2DCFDA to measure ROS. Cells were visualized under a fluorescence microscope. Representative photographs of bright field and H2DCFDA‐stained cells

We next examined whether ethanol‐transformed HPNE cells gained the phenotypes of cancer stem cells (CSCs) and express pluripotency maintaining markers (Figure 2). The formation of spheroids in suspension is the main characteristics of CSCs. After 6 months long exposure of HPNE cells to ethanol, HPNE cells demonstrated the features of cellular transformation, that is formed spheroids in suspension and colonies in soft agar (Figure 2A). Dimensions of spheroids and colonies formed by HPNE cells were greater with exposure of 100 mmol/L ethanol compared to 10 mmol/L ethanol. We next examined whether transformed HPNE cells gained the phenotype of CSCs by measuring stem cell markers (Figure 2B) and pluripotency maintaining factors (Figure 2C). Ethanol‐transformed HPNE cells expressed significantly higher levels of stem cell markers (CD24, CD44 and CD133) and pluripotency maintaining factors (Oct4, Sox2, cMyc and Klf4). We have validated the use of these markers and pluripotency maintaining factors for pancreatic CSCs.^26^, ^27^, ^28^ The induction of these genes was higher with 100 mmol/L of ethanol compared to 10 mmol/L ethanol. The expression of these genes was either low or not detected in HPNE cells. Reactive oxygen species (ROS) are generated during malignant transformation and are elevated in inflammatory environment. *N*‐acetylcysteine (NAC) is an aminothiol and synthetic precursor of intracellular cysteine and glutathione (GSH) and is thus considered an important antioxidant. Ethanol‐transformed HPNE cells generated ROS (Figure 2D). Furthermore, treatment of ethanol‐transformed HPNE cells with NAC inhibited intracellular ROS levels. These data suggest that ethanol can induce transformation of HPNE cells by converting them to CSC‐like phenotype.

### 3.3 SATB2 directly binds to promoters of Klf4, Oct4, cMyc, Sox2, Bcl‐2 and XIAP in ethanol‐transformed HPNE cells

**Figure 3:**
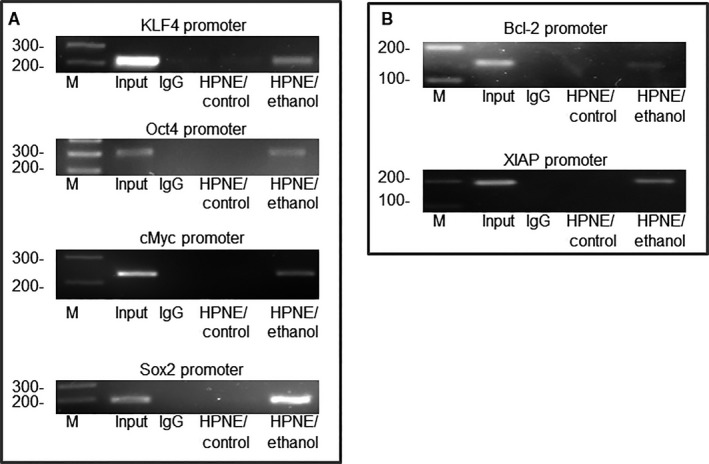
Binding of SATB2 to promoters of KLF4, Oct4, cMyc, Sox2, Bcl‐2 and XIAP. A–B, Chromatin immunoprecipitation (ChIP) assays were performed to examine the binding of the SATB2 to the promoters of KLF4, Oct4, cMyc, Sox2, Bcl‐2 and XIAP in HPNE/control and HPNE/Ethanol cells as described elsewhere.^47^, ^50^ ChIP‐derived DNA was quantified by 2% agarose gel electrophoresis

SATB2 is a transcription factor and thus can regulate various cellular functions by directly binding to target genes.^29^ Some of the target genes of SATB2 regulate pluripotency, self‐renewal, and cell survival. ChIP assay is commonly used to examine the binding of a transcription factor to the promoter regions of the target genes. We, therefore, investigated the binding of SATB2 to the promoters of potential gene targets in HPNE‐transformed cells. SATB2 can directly bind to promoters of KLF4, Oct4, cMyc, Sox2, Bcl‐2 and XIAP (Figure 3). These data suggest that SATB2 can directly bind to these genes and regulate their expression in ethanol‐transformed cells.

### 3.4 Knockdown of SATB2 by shRNA in ethanol‐transformed cells inhibits cell proliferation and colony formation

**Figure 4:**
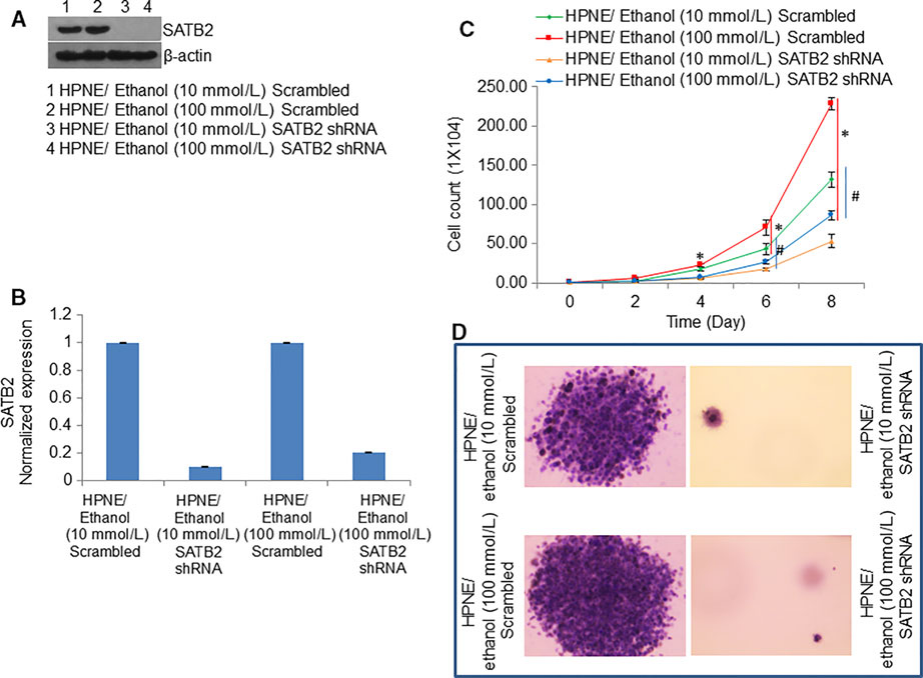
SATB2 shRNA inhibits cell proliferation and colony formation. A, Ethanol‐transformed HPNE cells (10 or 100 mmol/L ethanol for 6 mo) were transduced with either scrambled or SATB2 shRNA. The expression of SATB2 was confirmed by the Western blotting analysis. β‐actin was used as a loading control. B, RNA was isolated and q‐RT‐PCR was performed to measured the expression of SATB2 mRNA. Data represent mean ± SD. *, or # = significantly different from scrambled control group, *P* < .05. C, Cell proliferation was measured over 8 d. Data represent mean ± SD. *, #Significantly different from scrambled control group, *P* < .05. D, Colony formation. Ethanol‐transformed cells were seeded in Petri dishes. The number of colonies formed in 21 d was photographed.

To examine whether SATB2 in involved in in vitro cellular transformation, we knocked‐down the expression of SATB2 by shRNA in ethanol‐transformed HPNE cells which were exposed to 10 or 100 mmol/L ethanol for 6 months. Ethanol‐transformed HPNE cells were transduced with either scrambled or SATB2 shRNA lentiviral particles, and cell growth and colony formation were measured (Figure 4). Transduction of Ethanol‐transformed HPNE cells (HPNE/Ethanol [10 or 100 mmol/L]) with SATB2 shRNA viral particles inhibited the expression of SATB2 proteins and mRNA compared to that of HPNE/Ethanol (10 or 100 mmol/L)/Scrambled cells (Figure 4A and B).

We next examined the effects of inhibiting SATB2 on cell proliferation, and colony formation by ethanol‐transformed cells (Figure 4C and D). SATB2 shRNA inhibited cell proliferation, and colony formation in both ethanol‐transformed HPNE groups (HPNE/Ethanol [10 mmol/L]/SATB2 shRNA and HPNE/Ethanol [100 mmol/L]/SATB2 shRNA) compared to that of Scrambled groups (HPNE/Ethanol [10 mmol/L]/Scrambled and HPNE/Ethanol [100 mmol/L]/Scrambled). These data suggest that SATB2 can inhibit the ability of Ethanol‐transformed cells to proliferate and form colonies.

### 3.5 SATB2 inhibits the expression of stem cell markers in ethanol‐transformed cells

**Figure 5:**
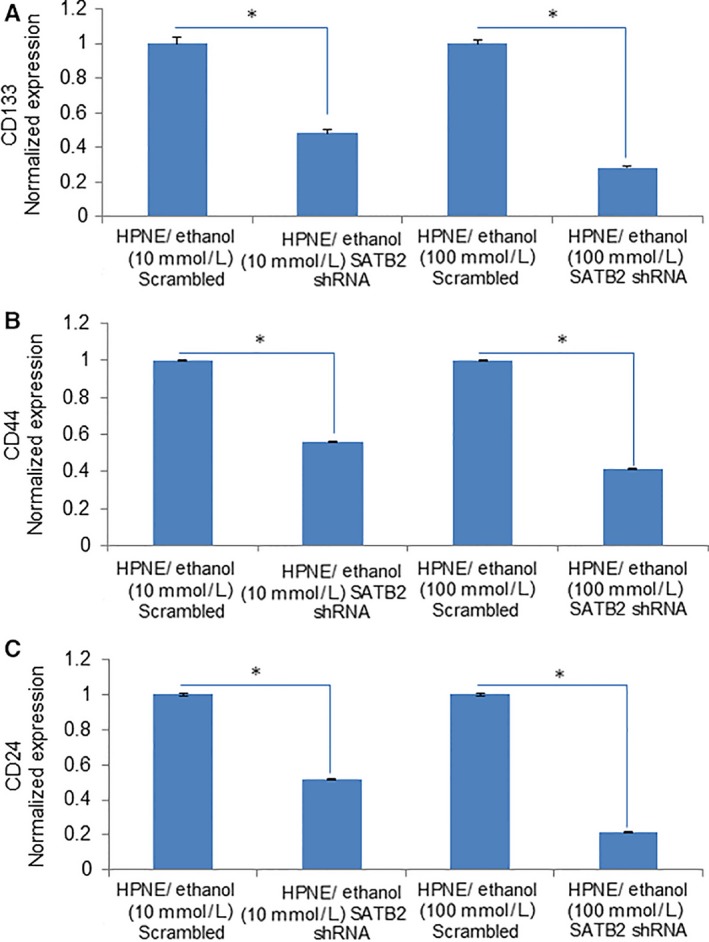
SATB2 inhibits the expression of stem cell markers in ethanol‐transformed cells. A‐C, Ethanol‐transformed human pancreatic normal ductal epithelial (HPNE) cells (HPNE/Ethanol [10 mmol/L], and HPNE/Ethanol [100 mmol/L]) were transduced with lentiviral particles expressing either scrambled or SATB2 shRNA. RNA was isolated, and the expression of CD133, CD44 and CD24 was measured by qRT‐PCR. Data represent mean ± SD. *Significantly different from scrambled control group, *P* < .05

Our data demonstrate that chronic exposure of HPNE cells with ethanol induces stem cell markers CD133, CD44 and CD24. We, therefore, sought to examine whether inhibition of SATB2 by shRNA attenuates the expression of these markers in ethanol‐transformed cells (Figure 5A‐C). SATB2 shRNA inhibited the expression of CD133, CD44 and CD24 in ethanol‐transformed cells (HPNE/Ethanol [10 mmol/L]/SATB2 shRNA, and HPNE/Ethanol [100 mmol/L]/SATB2 shRNA) compared to that of scrambled control groups. These data suggest that inhibition of SATB2 can result in suppressing stem cell population in ethanol‐transformed HPNE cells (HPNE/Ethanol).

### 3.6 SATB2 inhibits the expression of pluripotency maintaining factors in ethanol‐transformed cells

**Figure 6:**
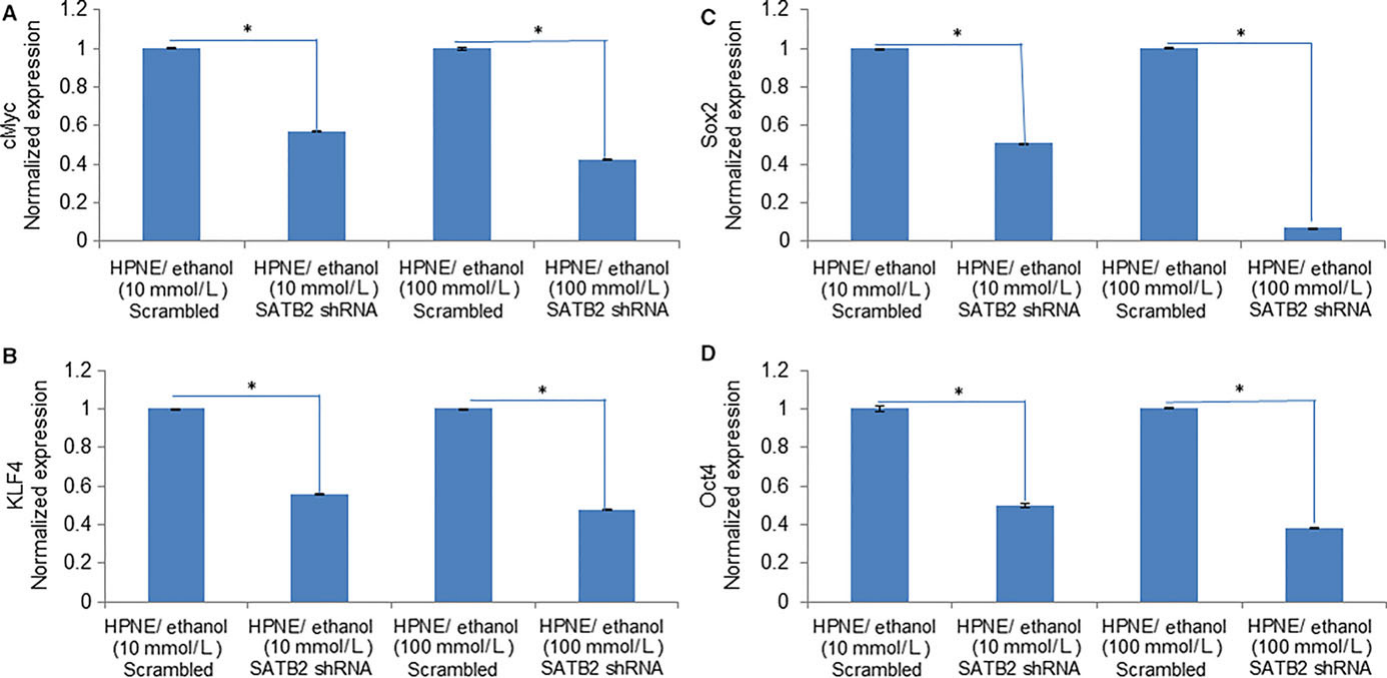
SATB2 inhibits the expression of pluripotency maintaining factors in ethanol‐transformed cells. A‐D, Ethanol‐transformed human pancreatic normal ductal epithelial (HPNE) cells (HPNE/Ethanol [10 mmol/L] and HPNE/Ethanol [100 mmol/L]) were transduced with lentiviral particles expressing either scrambled or SATB2 shRNA. RNA was isolated, and the expression of cMyc, KLF4, Sox2 and Oct4 was measured by qRT‐PCR. Data represent mean ± SD. *Significantly different from scrambled control group, *P* < .05

Since ethanol‐transformed HPNE cells demonstrated stemness by overexpressing pluripotency maintaining factors, we next sought to examine whether inhibition of SATB2 will attenuate the expression of cMyc, KLF4, Sox2 and Oct4 in ethanol‐transformed cells (Figure 6A‐D). SATB2 shRNA inhibited the expression of cMyc, KLF4, Sox2 and Oct4 in ethanol‐transformed HPNE cells (HPNE/Ethanol [10 mmol/L]/SATB2 shRNA, and HPNE/Ethanol [100 mmol/L]/SATB2 shRNA). These data suggest that SATB2 can regulate pluripotency and self‐renewal by regulating the expression of cMyc, KLF4, Sox2 and Oct4 in ethanol‐transformed HPNE cells (HPNE/Ethanol).

## 4 DISCUSSION

The proposed studies provide, for the first time, the carcinogenic effects of alcohol on HPNE cells. We have provided ample evidence that during ethanol‐induced cellular transformation, CSCs/progenitor cells are developed which may play a significant role in pancreatic carcinogenesis. The current findings enhance our understanding of the signalling pathways which regulate cellular transformation and examine how chronic exposure of HPNE cells to ethanol induces stem cell markers, and pluripotency maintaining factors resulting in the generation of cancer stem‐like cells. The conversion of HPNE cells to cancer stem‐like cells by ethanol pointing a pathophysiological role of alcohol abuse in pancreatic carcinogenesis. The conversion of HPNE cells to stem‐like cells by ethanol will be a significant event for those individuals who are addicted to alcohol consumption.

Alcohol abuse is a risk factor for pancreatic cancer. Genetic and other environmental factors like cigarette smoke may further potentiate the adverse effects of alcohol. On the basis of our findings, we hypothesized that ethanol toxicity induces HPNE cell transformation and may induce or enhance, alone or with other factors, pancreatic carcinogenesis by modulating the expression of SATB2. We recently showed that up‐regulation of SATB2 in HPNE cells was sufficient to induce malignant transformation and those transformed cells gained the phenotypes of CSCs by expressing CSC markers and pluripotency maintaining factors.^24^, ^30^ Chronic exposure of HPNE cells to 100 mmol/L ethanol caused ~300‐fold increase in CD133 expression, whereas knockdown of SATB2 resulted in a moderate, ~2.5‐fold reduction in CD133 levels. These data suggest the possibility of other transcription factors/genes that might contribute to regulation of ethanol‐dependent transformation processes. Furthermore, SATB2‐transformed cells also showed high levels of Cyclin D1 and Bcl‐2, which are required for cell cycle progression and cell proliferation.^24^, ^30^

Epidemiological data strongly suggest that the heavy drinking increases the risk for pancreatic cancer.^6^, ^10^, ^31^, ^32^ High alcohol intake was associated with a higher risk of pancreatitis.^33^, ^34^, ^35^ Diabetic patients and heavy smokers who consume excessive alcohol demonstrate a high risk of developing pancreatic cancer. In compared to pancreatitis, the role of alcohol consumption remains less clear in pancreatic cancer. A low to moderate alcohol consumption does not appear to be associated with pancreatic cancer risk, and only chronic heavy drinking increases the risk of pancreatic cancer. Several factors have been demonstrated to contribute towards the development of alcohol‐associated cancer.^10^, ^36^, ^37^, ^38^, ^39^ Evidence suggests that the effect of alcohol is modulated by polymorphisms in genes encoding enzymes for ethanol metabolism (eg alcohol dehydrogenases, aldehyde dehydrogenases and cytochrome P450 2E1), folate metabolism and DNA repair. Several events including a genotoxic effect of acetaldehyde increased oestrogen concentration, a role as the solvent for tobacco carcinogens, production of ROS and nitrogen species, and changes in folate metabolism have been implicated. Daily consumption of more than 80 g alcohol (more than five to six drinks) with smoking increases the risk of developing cancers by a factor of 50 or more.^40^, ^41^ In support of our study, it was demonstrated that chronic alcohol consumption promotes intestinal tumorigenesis and tumour invasion in genetically susceptible mice.^15^ During ethanol metabolism, ethanol is oxidized to acetaldehyde by ADH or CYP2E1.^42^, ^43^ If the patients consume alcohol, acetaldehyde concentrations in the stomach increase 6.5‐fold.^44^ In addition to the acetaldehyde generated by cellular enzymes or gastrointestinal bacteria, considerable amounts of acetaldehyde are present in certain alcoholic beverages and cigarette smoke.^44^ Based on these findings it appears that ethanol and its metabolites are the potential human carcinogens.

SATB2 may act as a master regulator of pluripotency and self‐renewal because SATB2 binding sites are present in the promoter regions of KLF4, Oct4, cMyc and Sox2.^45^, ^46^, ^47^, ^48^ Similar to pancreatic CSCs, we have found that SATB2 is highly expressed in colorectal and breast CSCs.^24^, ^30^ Interestingly, the expression of SATB2 was absent or very low in HPNE cells, mammary epithelial cells and colorectal epithelial cells.^20^, ^24^, ^30^ Furthermore, overexpression of SATB2 in normal epithelial cells resulted in malignant transformation, suggesting an oncogenic role of SATB2 in various cancers. In the present study, ethanol‐transformed cells express high levels of SATB2 which was associated with generation of ROS. At present the basis of SATB2 induction by chronic ethanol exposure to HPNE cells is not clear. It is possible that chronic ethanol exposure of HPNE cells causes ROS production (an inflammatory environment) which in turn induces SATB2 expression and malignant transformation. Alternatively, other oncogenes are simultaneously amplified to sustain‐transformed phenotypes of CSCs, and high level of ROS is an outcome of transformation.

In conclusion, our data demonstrate that chronic ethanol exposure of HPNE cells induced malignant transformation, generated ROS, and these transformed cells gained the phenotype of CSCs by expressing SATB2, stem cell markers, and pluripotency maintaining factors. These findings are very significant for human health because alcohol can initiate or promote signalling events, either alone or in combination with other unknown factors, which could result in the development of pancreatic cancer. Further studies are underway in our laboratory to fully understand the pathological significance of alcohol toxicity in mouse models of pancreatic cancer. Overall, precautions should be taken when alcohol is consumed continuously for a longer period.

## CONFLICTS OF INTEREST

All the authors have declared that no conflict of interest exist.

## AUTHOR CONTRIBUTIONS

WY and YM performed the experiments, analysed the data and wrote the manuscript. SS, and RKS designed the study and contributed reagents. All authors read and approved the manuscript.
